# High-Temperature Hot Air/Silane Coupling Modification of Wood Fiber and Its Effect on Properties of Wood Fiber/HDPE Composites

**DOI:** 10.3390/ma10030286

**Published:** 2017-03-13

**Authors:** Feng Chen, Guangping Han, Qingde Li, Xun Gao, Wanli Cheng

**Affiliations:** 1 Key Laboratory of Bio-based Material Science and Technology (Ministry of Education), Northeast Forestry University, Harbin 150040, China; c.f84@hotmail.com (F.C.); guangping.han@nefu.edu.cn (G.H.); liqingde2017@hotmail.com (Q.L.); gaoxunbeihuauniversity@hotmail.com (X.G.); 2 Liaoning Forestry Vocational-Technical College, Shenyang 110101, China

**Keywords:** high-temperature hot air, modification mechanism, silane coupling agent, mechanical properties, interfacial compatibility, surface treatment, composite material

## Abstract

The surfaces of poplar wood fibers were modified using high-temperature hot air (HTHA) treatment and silane coupling agent. The single factor test was then used to investigate the performances (e.g., the change of functional groups, polarity, cellulose crystallinity, and thermal stability) of modified poplar wood fibers (mPWF) through Fourier transform infrared spectrometry, X-ray diffraction and thermo-gravimetric analysis for the subsequent preparation of wood-plastic composites (WPCs). The effect of HTHA treatment conditions—such as temperature, inlet air velocity, and feed rate—on the performances of WPCs was also investigated by scanning electron microscopy and dynamic mechanical analysis. The main findings indicated that HTHA treatment could promote the hydration of mPWF and improve the mechanical properties of WPCs. Treatment temperature strongly affected the mechanical properties and moisture adsorption characteristics of the prepared composites. With the increase of treated temperature and feed rate, the number of hydroxyl groups, holocellulose content, and the pH of mPWF decreased. The degree of crystallinity and thermal stability and the storage modulus of the prepared composites of mPWF increased. However, dimensional stability and water absorption of WPCs significantly reduced. The best mechanical properties enhancement was observed with treatment temperature at 220 °C. This study demonstrated the feasibility for the application of an HTHA treatment in the WPC production industry.

## 1. Introduction

Derived from wood or agricultural materials—such as kenaf, jute, hemp, flax, or other natural resources—natural fibers have been widely used as reinforced materials for plastics [1]. Water evaporation in natural fibers leads to the emergence of pores during the compounding process, which can result in inferior performances of wood-plastic composites (WPCs). Thus, the natural fibers must be pretreated to control moisture content at 1% to 3% [2]. However, the natural fibers can still be prone to agglomeration, due to the formation of hydrogen bonds between cellulose molecules that contain many hydroxyl groups and thus cause uneven dispersion during mixing [3,4]. Moreover, the majority of polar (hydrophilic) natural fibers are not compatible with non-polar (hydrophobic) substances, due to the existence of hydroxyl groups on the cellulose and hemicellulose of natural fibers. Therefore, it can result in poor interfacial compatibility between the natural fibers and thermoplastics in WPCs [5].

Wood fibers must be thoroughly pre-treated to obtain good dispersion [6]. Heat treatment method—e.g., drying pretreatment, high-temperature pressurized steaming pretreatment, or normal-temperature pressurized steaming pretreatment—is the conventional pretreatment method. Lanjie Li et al. researched the influence of the oven drying method on PE/pine powder composite materials, the results showed that drying the pine powder at 105 °C for 7 h could provide WPCs with better mechanical properties [7]. However, the effect of the drying modification mechanism on the mechanical properties has not been further studied. Moreover, the commonly used drying pretreatments—including oven drying, impinging stream drying, flash tube drying, etc. [8,9]—can still lead to wood fiber agglomeration and poor uniformity of the final moisture content and may not be appropriate for WPC production. Ayrilmis N. et al. aimed to enhance the dimensional stability of flat-pressed wood plastic composites containing fast growing wood fibers by a thermal-treatment method [10]. The wood fibers were treated at three different temperatures (120, 150, or 180 °C) for 20 or 40 min in a laboratory autoclave. The results showed that thickness swelling and water absorption of the WPC panels significantly decreased with increasing the treatment temperature and time, and the flexural properties and internal bond strength were more seriously affected by the treatment. Yanjun Li et al. treated the bamboo at high temperature [11]. The results showed that the contents of holocellulose decreased from 76.14% to 72.14% with the increase of treatment temperature from 100 °C to 180 °C and the slightly increased of lignin contents, while the change of the mechanical properties of bamboo/high-density polyethylene (HDPE) composites is not obvious. Xun G. et al. investigated high temperature and pressurized steaming/silane coupling co-modification on the properties of wood fiber/HDPE composites. These scholars asserted that the thermal stability, crystallinity, and dynamic thermal-mechanical properties of the composites were improved by high-temperature heat treatment [12]. However, these methods usually have several disadvantages such as long treatment time, complexity, and low production.

A low-energy consuming and fast heat treatment method at a high temperature that provides a good dispersion, high product quality, and low moisture content to wood fibers will be one of the key technologies for WPC development. Few studies have examined the impact of a high-temperature hot air treatment on the quality of wood fibers, their dispersion in the plastic matrix, and the mechanical properties of WPCs. Compared to other heat treatments, impulse and cyclone heat treatment method can easily remove free water and most bound water of materials, due to the long residence time of material in the cyclone unit and acceleration and deceleration alternating in the impulse unit, which increases the mass and heat transfer efficiency. Moreover, high-temperature hot air can improve the fiber quality, which leads to a much lower number of free hydroxyl groups on the surface, a decreased polarity, a higher surface roughness, and a higher crystallinity of the cellulose.

This study explored modification mechanism of high-temperature hot air treatment and silane coupling agents on poplar fibers. The effect of the different treatment parameters on the fiber quality, the dispersion of wood fibers in the plastic matrix, chemical composition, crystallinity, holocellulose content, pH, and the thermal stability of fibers was determined to obtain the final product that could be used in WPCs. Meanwhile, it provides a new modification method to enhance the properties of HDPE/wood fiber composites.

## 2. Material and Methods

### 2.1. Raw Materials

HDPE (5000S resin, density 0.954 g·cm^−3^, melt flow index 0.7 g/10 min, crystallinity percent 85%, and mol. wt. 3.0 × 10^5^) from Daqing Petrochemical Co. (Daqing, China) was used in the study as the matrix. Harbin Yongxu Wood-based Panel Co., Ltd. (Heilongjiang, China) supplied the poplar wood veneers with sizes ranging from 50 cm to 100 cm in length, 30 cm to 50 cm in width, and 0.4 cm to 0.6 cm in thickness. Using a wood fiber mill (FY600, Fuyang Energy Technology Co., Ltd., Xuzhou, China), the poplar wood veneers were smashed and milled into poplar wood fibers with particle sizes ranging from 60- to 80-mesh [13]. The characteristics of poplar wood fibers after milling are shown in Table 1. 

The silane coupling agent (A-187: 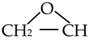
CH_2_OCH_2_CH_2_CH_2_(CH_2_)_3_Si(OCH_3_)_3_) with a boiling point of 290 °C (Quanxi Chemical Co., Ltd., Nanjing, China), industrial-grade paraffin (density 0.95 g/cm^3^, mol. wt. 3500) as lubricant, MgCl_2_, NaNO_2_, NaCl, KCl, Na_2_SO_4_, acetone, ethanol, and glacial acetic acid were used as raw materials in this study.

### 2.2. High-Temperature Hot Air Treatment/Silane Coupling Agent Modification of Poplar Wood Fibers

Poplar wood fibers were treated in a high-temperature environment with an impulse and cyclone heat treatment device (MQG-50, Jianda Equipment Co., Ltd., Changzhou, China). The optimal condition for high-temperature hot air (HTHA) treatment was selected through preliminary tests. The wood fibers were first subjected to high-temperature hot air generated using a heat generator, a screw feeder, and an induced draft fan at different temperatures (160, 180, 200, 220, 240 °C), inlet air velocity (9, 10, 11, 12, 13 m/s), and feed rate (90, 105, 120, 135, 150 kg/h). The silane coupling agent was then mixed with the HTHA-treated poplar wood fibers at a mass ratio of 3:100 in a high-speed mixer (SHR-10A, Tonghe Plastic Machinery Co., Ltd., Zhangjiagang, China). Afterwards, the modified poplar wood fibers were re-processed through the HTHA treatment device at low temperature (105 °C), air velocity (6 m/s), and feed rate (50 kg/h) for 5 min to decrease the moisture content to below 3%.

### 2.3. Preparation of Wood Fiber/HDPE Composites

Modified poplar wood fibers (50 wt. %), HDPE (48 wt. %), and paraffin [14] (2 wt. %) were mixed and stirred in a high-speed mixer. The resultant uniform mixture was then placed into an SJSH30/SJ45 two-stage plastic extruder [15] (Nanjing Rubber & Plastic Mechanic Co. Ltd., Nanjing, China). After granulation and extrusion molding, the standard specimens were prepared for impact, flexural, and tensile testing. There were six specimens in each group with the dimensions of 80 × 10 × 4 mm^3^ (impact tests), 80 × 13 × 4 mm^3^ (flexural tests), and 165 × 20 × 4 mm^3^ (tensile tests).

### 2.4. Characterization of Wood Fibers 

#### 2.4.1. Fourier Transform Infrared Spectrometry Procedure and Analysis

Using the KBr disc technique (1 mg of sample powder/100 mg of KBr), the Fourier transform infrared spectrometry measurement was performed with a Nicolet Nexus 6700 FTIR spectrometer (American Thermo Fisher Scientific company, Waltham, MA, USA) at a resolution of 4 cm^−1^, 32× scans, and a scanning range of 4000 cm^−1^ to 600 cm^−1^. Three replicate measurements were recorded for each condition [16].

#### 2.4.2. Wide-Angle X-ray Diffraction (WXRD)

A D/MAX 2200 X-ray diffractometer (Rigaku Corporation, Tokyo, Japan) was used to measure the WXRD patterns of the fiber samples before and after modification [17]. Prior to the measurement, the samples were placed onto the supporter and pressed compactly. Over the angular range of 2θ = 5° to 40° and a step size of 5°/min, the WXRD data was generated by a diffractometer with Cu Kα radiation (λ = 1.542 Å) at 40 kV and 30 mA. The degree of crystallinity, or crystallinity index (*CI* %), was evaluated for each sample using Equation (1),

(1) $$CI\%=(A_{c}/A_{a})\times 100$$

where *A*_c_ is the area of the crystalline reflection and *A*_a_ is the area subtending the whole diffraction profile. The WXRD jade software (MDI JADE 6.5, Materials Data, Inc., Livermore, CA, USA) was adopted to calculate the diffraction peaks (002), the crystalline reflection, and the area subtending the whole diffraction profile.

#### 2.4.3. Holocellulose Extraction

According to the Chinese National Standard (GB/T 2677-1995) [18], the sodium chlorite method was used to determine the holocellulose content. In the experiment, a mixture of toluene and ethanol was used to extract the substance from the modified poplar wood fibers, and then sodium chlorite and glacial acetic acid were added to the extracted substance for the delignification treatment. After washing, the extracted substances were dried in a drying oven (DHG-9030A, Shanghai Precision Instrument Co., Ltd., Shanghai, China) at 103 ± 2 °C. The sample was removed from the drying oven and weighed every two hours. When the mass change was less than 0.002 g, the absolute drying status of the substances was determined. The holocellulose content was calculated based on the oven-dried weight.

#### 2.4.4. The pH Value

Three grams of wood fibers were weighed using an analytical electronic balance (range: 0–210 g, precision 0.1 mg) (model AE100, Mettler Instrument, Highstown, NJ, USA) and placed in a beaker. Then, 30 mL of distilled water was added to boil for 2 min and cool to room temperature. The mixture was further thoroughly stirred. The test for pH value of the modified poplar wood fibers was carried out based on GB/T 6043-2009 standard [19].

#### 2.4.5. Thermogravimetric Analysis (TGA)

A TA 309F3 thermal gravimetric analyzer (TA Instruments, New Castle, DE, USA) was used to measure the thermal decomposition of modified poplar wood fibers during the heating process. The parameters were: a heating rate of 10 °C/min, a nitrogen flow rate of 30 mL/min, a temperature range of 20–600 °C, and a sample amount of 5 mg [20].

### 2.5. Characterization of the Prepared Wood Fiber/HDPE Composites

#### 2.5.1. Mechanical Properties

The specimens with the typical dimensions of 165 × 20 × 4 mm^3^ for tensile testing were measured using an RGT-20A electronic mechanics testing machine WDW-50 electronic mechanics testing machine (Changchun Kexin Instrument Equipment Co., Ltd., Changchun, China) according to ASTM D790 [21]. A crosshead speed of 10 mm/min and a gauge length of 64 mm were used for the test. Flexural testing was conducted from specimens with a dimension of 80 × 13 × 4 mm^3^ under the three-point bending test using the same Universal Testing Machine. A crosshead speed of 5 mm/min and a span length of 64 mm were used for the test. The impact strength was measured from specimens with a dimension of 80 × 10 × 4 mm^3^ using a XJ-50G impact tester (Chengde Precision Testing Machine Co. Ltd., Chengde, China) according to ASTM D5628 [22]. Each group of samples was simultaneously measured five times, and the mean value of five parallel measurements along with corresponding was taken to reflect the experiment result.

#### 2.5.2. Dynamic Mechanical Analysis Test

Dynamic mechanical analysis (DMA) of wood fiber/HDPE composites was performed using a TA Q800 analyzer (TA Instruments Inc., New Castle, DE, USA). The testing temperature ranged from 40 °C to 130 °C and the heating rate was 3 °C·min^−1^. Three replicates with dimensions of 35 mm in length, 12 mm in width, and 3 mm in thickness were performed for each sample.

The interfacial bonding between the modified poplar wood fiber and the polymer matrix can be evaluated using the adhesion factor (*A*), which is determined from DMA data at 40 °C based on the study of Kubat et al. [23] by using Equation (2):

(2) $$A\left(\mathrm{MPa}\right)=\left(1/\left(1-V_{f}\right)\right)\left(\tan\delta_{c}/\tan\delta_{m}\right)-1$$

where c and m subscripts represent composite and matrix, respectively, and *V*_f_ is the fiber volume fraction. A low value of *A* is an indicator of good adhesion or a high degree of interaction between the two phases and vice versa. 

#### 2.5.3. Scanning Electron Microscopy (SEM)

A QuanTa-200 Environmental Scanning Electron Microscope (FEI Company, Eindhoven, The Netherlands) was used to observe morphologies of wood fiber/HDPE composites. The magnification was 300×. The samples were taken from the tensile dumbbell test. The samples were frozen in liquid nitrogen and fractured to obtain a well-defined fiber/matrix interface. The samples were spray-coated with gold prior to the observation to enhance the surface conductivity, and observed under SEM at an acceleration voltage of 15 KV [24]. 

#### 2.5.4. Adsorption Measurements

Sixteen specimens with the dimensions of 10 × 10 × 4 mm^3^ from each of the material types were randomly selected and numbered. First, the wood-plastic composite material was cut into the samples with above dimensions using small sample preparation equipment (XXZ-II, Chengde Jinjian Test Equipment Co., Chengde, China). Then, the burrs on the edge of the samples were removed with 800 mesh sandpaper (M10, Lifeng Sandpaper Co., Foshan, Guangdong, China). Eight specimens were placed in a DHG-9030A oven dryer (Shanghai Shuangxu Electronics Co., Shanghai, China) at 103 ± 2 °C to reach the absolute dry state for adsorption test. The remaining eight specimens were placed in distilled water until the saturation state for the desorption test. All of the samples were conditioned to reach equilibrium at a relative humidity of 33%, 66%, 75%, 85%, and 93%, respectively, in different saturated saline solutions of desiccators [25]. The initial weight of all specimens was measured. The equilibrium moisture content (EMC) of each specimen was calculated based on the oven-dry weight. The following Equation (3) was used to measure the EMC, which was accurate to 0.01 g,

(3) $$EMC\;\left(\%\right)=\frac{W_{1}-W_{0}}{W_{0}}\times 100$$

where *EMC* (%) is the equilibrium moisture content of the sample, *W*_1_ (g) is the sample mass after adsorption and desorption tests, and *W*_0_ (g) is the absolute dry mass of the sample.

## 3. Results and Discussion

### 3.1. Fourier Transform Infrared Spectrometry (FTIR) Analysis

**Figure 1:**
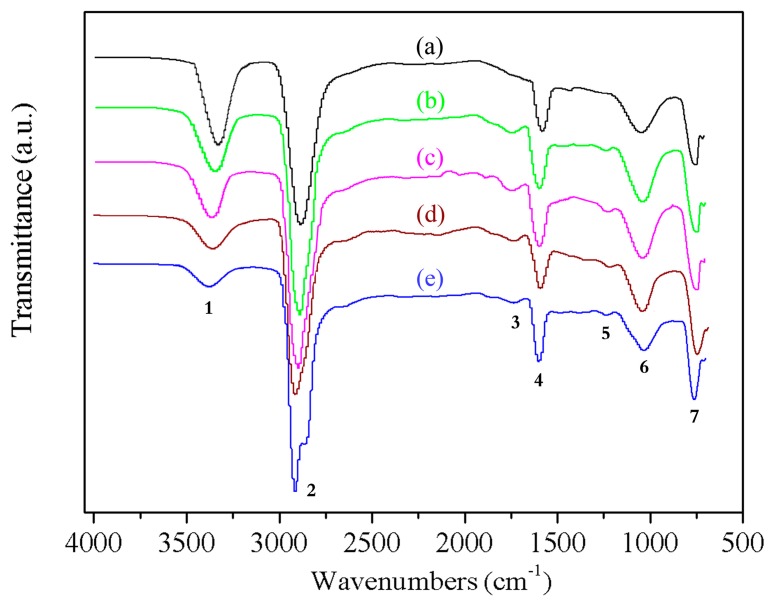
Comparison of FTIR spectra obtained for modified poplar wood fibers under different treatment temperatures; (**a**) *T* = 160 °C; (**b**) *T* = 180 °C; (**c**) *T* = 200 °C; (**d**) *T* = 220 °C; (**e**) *T* = 240 °C.

The FTIR spectra attribution results for the wood fibers modified through high-temperature hot air and silane coupling agent are shown in Table 2 and Figure 1. A strong hydrogen bonded (O–H) stretching absorption is seen at 3350 cm^−1^ (1) and a prominent C–H stretching absorption is seen around 2890 cm^−1^ (2). The peaks in the fingerprint are assigned: 1730 cm^−1^ (3) for unconjugated C=O in xylan (hemicellulose), 1600 cm^−1^ (4) for benzene ring carbon skeleton stretching (lignin), 1240 cm^−1^ (5) for benzene-oxygen bond stretching vibration in lignin and acyl-oxygen bond (CO-OR) stretching vibration in hemicellulose, 1030 cm^−1^ (6) for aromatic skeletal and C–O stretch. The characteristic adsorption peak at 767 cm^−1^ (7) resulted from the Si–O stretching vibrations formed by the self-polymerization of the silanol groups [26]. 

Figure 1 shows the comparison of the FTIR spectra obtained for modified poplar wood fibers (mPWF) under different treatment temperature. The high-temperature hot air (HTHA) treatment showed little impact on the chemical composition of poplar wood fiber, while it had a large impact on the absorption intensity of functional groups. The absorption intensity of the characteristic peaks of the samples treated under different temperature did not change noticeably. However, the absorption intensity of the –OH stretching vibration peak at 3350 cm^−1^ decreased obviously with the increase of the temperature of the HTHA treatment. This indicated that the number of free hydroxyl groups decreased with higher temperatures. The silanol groups generated by the hydrolyzed silane can form covalent Si–O–C bonds with the poplar fibers. The presence of both bonds indicates that the coupling agent was grafted onto the poplar fibers via a co-polymerization reaction. This chemical reaction affected the surface composition of the poplar fibers to some extent. As the temperature increased, the adsorption intensity of methyl (–CH_3_) and methylene (–CH_2_) stretching vibration peaks around 2890 cm^−1^ also decreased. This result revealed that a higher treatment temperature accelerated the molecules’ decomposition and esterification within the fiber. The peak at 1730 cm^−1^ was attributed to the stretching vibration of the non-conjugated carbonyl group (C=O) of xylan, which was the main characteristic peak of hemicellulose and different from other components. With the increase of temperature, the adsorption intensity of the C=O stretching vibration peak decreased, which showed that the higher temperature and moist environment in hot air could make the acetyl group hydrolyze into acetic acid and reduce the number of hydrophilic C=O functional groups. The peaks around 1600 cm^−1^ were attributed to the carbon skeleton vibration of benzene rings, which were the characteristic absorption peaks of lignin. For the sample treated at 160 °C and 200 °C, the intensity did not vary much. However, the intensity was reduced significantly at 240 °C, which indicated that the lignin in fiber had decomposed below 240 °C. The peaks around 1240 cm^−1^ were the characteristic absorption peaks of hemicellulose and lignin. These peaks diminished gradually as the temperature increased, which indicated that higher temperatures could promote hemicellulose deacetylation and ultimately decompose it into monosaccharides [27].

### 3.2. Wide-Angle X-ray Diffraction (WXRD)

**Figure 2:**
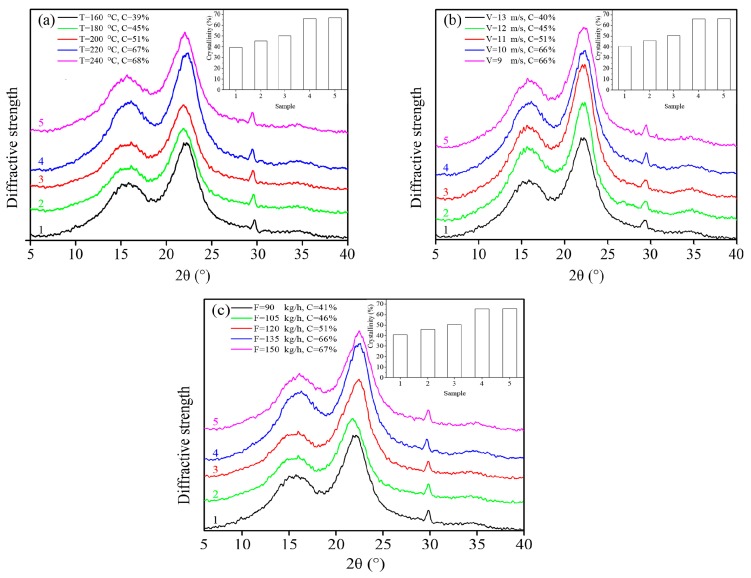
WXRD patterns obtained for poplar wood fibers by high-temperature hot air treatment under different treatment conditions. (**a**) Different temperatures with constant inlet air velocity 11 m/s and feed rate 120 kg/h; (**b**) Different inlet air velocity with constant temperature 200 °C and feed rate 120 kg/h; (**c**) Different feed rate with constant temperature 200 °C and inlet air velocity 11 m/s.

The crystallinity of wood materials was one of the primary factors that affected the mechanical properties. Figure 2 illustrates the WXRD patterns obtained for the un-modified and modified wood fibers. As shown in Figure 2, the (002) diffraction peaks recorded for the un-modified and modified wood fibers were both centered at approximately 22° (ranging from 21.32° to 22.88°). This result indicated that the modification process resulted in a limited change of the crystalline regions of wood fibers and the minimal effect on the distance between crystal layers [28]. However, high-temperature hot air (HTHA) treatment had a profound impact on the crystallinity of wood fibers.

As shown in Figure 2a, the crystallinity of modified poplar wood fibers (mPWF) was from 39% to 68%, indicating that increasing the HTHA treatment temperature had a stronger impact on the amorphous region of the fiber. This could have been due to the fact that, through the HTHA treatment, the “bridging reaction” between the hydroxyl groups of microfibrils in the amorphous region occurred, the hydroxyl groups dehydrated and formed ether bonds, microfibrils were arranged in the amorphous region, and the crystallinity of cellulose was then increased.

As shown in Figure 2b, the crystallinity of the treated specimen cellulose increased from 40% to 66% with the decrease of inlet air velocity. When the inlet air velocity was lower, the retention time of the poplar fiber in the drier increased, which led to the degradation of microfibers in the non-crystallizing area of cellulose, and thus the relative crystallinity of cellulose increased. 

As shown in Figure 2c, the cellulose crystallinity was from 41% to 67%. A high feed rate increased the charging frequency and collision chance of wood fibers, which increased the exposed area of fibers to high-temperature hot air which enhanced the cellulose crystallinity.

### 3.3. Effect of the High-Temperature Hot Air Treatment on the Holocellulose Content of the Wood Fibers

**Figure 3:**
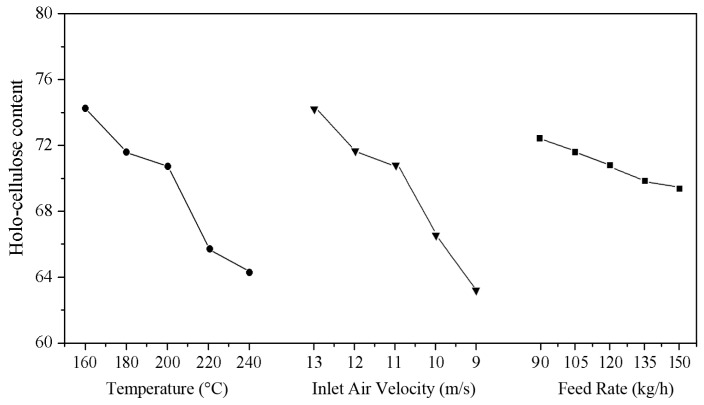
Effect of high temperature, inlet air velocity, and feed rate on the holocellulose content of wood fibers.

Figure 3 shows the holocellulose content of wood fibers as a function of air temperature, inlet air velocity, and feed rate. The holocellulose content of the wood fibers was found to decrease with increasing temperature and decreasing air velocity because the high-temperature and long retention time could remove the acetyl group of the semi-cellulose and induce the formation of acetic acid. Under the resulting acidic conditions, the glycoside bond breaks and a more thorough degradation occurs, whereas the lignin only experiences a slight softening rather than degradation due to its higher thermal stability [29]. At a high feed speed, the carrier tape rate of the wood fiber was high in the air flow. The increasing humidity of the hot air flow and long retention time promoted formation of acetic acid, which hydrolyzed the glucose unit into short chain structures, and thus the holocellulose content was reduced.

**Figure 4:**
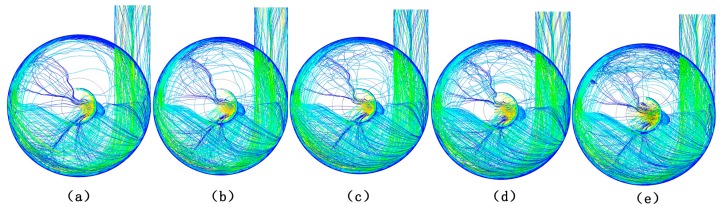
The flow field of the equipment with different feed rate by Fluent software. (**a**) Feed rate at 90 kg/h; (**b**) feed rate at 105 kg/h; (**c**) feed rate at 120 kg/h; (**d**) feed rate at 135 kg/h; (**e**) feed rate at 150 kg/h.

20 kg of poplar wood fibers were loaded before the test, the feed switch was opened and the timing was started, and the time for the complete removal of the poplar wood fibers was taken as termination time of the timing. The heat treatment time of different treatment conditions was as shown in Table 3. As can be seen from the table, the lower the air velocity and higher the feed rate was, the longer the rotation time and the distance of the fibers stayed in this section. Figure 4 showed the flow field of the equipment with different feed rates by Fluent software (Fluent 14.5, ANSYS Inc., Canonsburg, PA, USA). When the poplar fibers entered into the cyclone unit of HTHA treatment device tangentially, the fibers being separated from the hot air by the centrifugal force were rotated along the inner wall until they were brought into the bottom of the equipment. As upward an vertical tube at the bottom of the device impeded the air movement to a certain extent, resulting in the formation of vortex at the entrance of tube, sedimentary fibers moved around the vortex with the airflow. When the feed rate increased, more poplar fibers were brought into the bottom of device and the reverse flow caused by collision of fibers formed, so that the fibers could not enter the upward vertical tube successfully. The fibers had long retention time until being conveyed by airflow, thus reducing the holocellulose content. Thermal stability of hemicellulose was poor and degradation occurred under the condition of 180 °C [30].

### 3.4. Effect of the High-Temperature Hot Air Treatment on pH of Wood Fibers

**Figure 5:**
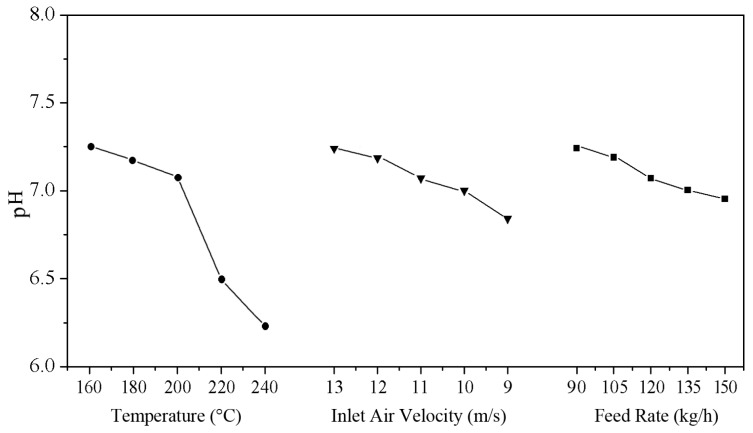
Effect of the high-temperature hot air treatment on the pH of the wood fibers.

The results of pH of modified poplar wood fibers (mPWF) under various high-temperature hot air (HTHA) treatments are shown in Figure 5. After HTHA treatments, the pH of the mPWF decreased remarkably. The pH of the treated fibers under 240 °C was reduced to 6.15 from 7.25 of 160 °C sample. Moreover, the pH of the mPWF decreased with increasing temperature, air velocity, and feed rate of the treatment. This result indicated that the acidic properties of mPWF increased after HTHA treatment. During the treatment, the semi-cellulose with its comparatively poor heat resistance partially degrades, losing its acetyl group, resulting in the formation of acetic acid [31]. Table 4 shows the results of humidity of air flow measured by high-temperature water vapor humidity analyzer (M7873H2O, ADEV Automation System Equipment Co., Shanghai, China). The anemometer was pulled out and a high-temperature water vapor humidity analyzer was put into the inspect hole to detect the air humidity when discharging was stable. The results indicated that the lower the inlet air speed, the greater the feed rate, and the greater the relative humidity in hot air. The long residence time, which can be seen from Table 3, causes the combined water and chemical water to separate from the fiber, resulting in increased relative humidity in the hot air. The higher temperatures could promote hydroxyl groups in cellulose to oxidize into aldehyde or carboxyl which increase the acidity of aqueous solution. The higher feed rate and lower air velocity made the fibers easy to hydrolyze hemicelluloses, losing its acetyl group, resulting in the formation of acetic acid in a higher humidity environment. During the modification process, an increase in temperature or feed rate leads to the generation of a higher amount of acidic substances. 

### 3.5. Thermal Analysis

**Figure 6:**
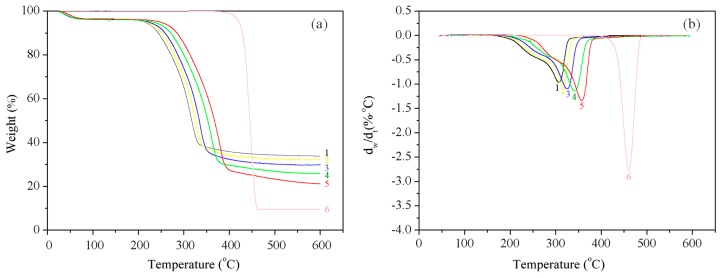
(**a**) TG and (**b**) DTG curves obtained for the unmodified and modified wood fibers; (1) un-modified fibers; (2) *T* = 180 °C; (3) *T* = 200 °C; (4) *T* = 220 °C; (5) *T* = 240 °C; and (6) HDPE.

Figure 6a,b shows the themogravimetric (TG) and derivative thermal gravimetric (DTG) curves for the modified wood fibers, respectively. Figure 5a shows that when the temperature increased from room temperature to 600 °C, the pyrolysis of the modified poplar wood fibers (mPWF) underwent four stages. In the first weight loss (23–90 °C), desorption of water or softening and melting of some wax components in the samples mainly happened. Then in the second stage (90–210 °C), mainly tiny weight loss of samples on account of depolymerization and glass transition of fibers occurred. The third stage (210–400 °C) was the main pyrolysis stage of fiber. Hemicellulose and cellulose were pyrolyzed into small molecule- and macromolecule-condensable gas volatilization, which caused apparent weightlessness. When the temperature continued to rise, the final generation of part of the carbon and ash occurred.

With the increasing temperature of high-temperature hot air, the wood fibers exposed to the high-temperature hot air showed a higher pyrolysis peak temperature, i.e., a better heat stability. When subjected to high-temperature hot air, parts of the structure of the hemicellulose underwent pyrolysis, which resulted in a slight increase in lignin content. Notably, the methoxy group, one of the characteristic functional groups of lignin, is relatively stable; therefore, more energy was needed to break the molecular structure of lignin. Furthermore, the hydroxyl groups in the cellulose can get oxidized into relatively stable aldehyde, carbonyl, and carboxyl groups to form oxidized cellulose. Therefore, a higher temperature of HTHA resulted in a better heat stability [32].

### 3.6. Effect of High-Temperature Hot Air Treatment on Mechanical Properties of Wood Fiber/HDPE Composites

**Figure 7:**
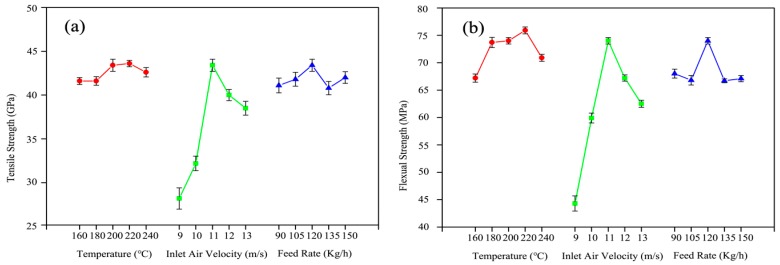

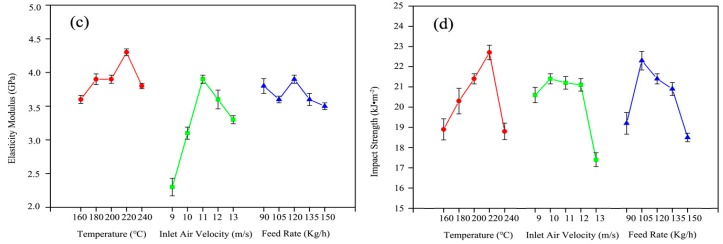
Mechanical properties of WPCs by modification of high-temperature hot air and silane coupling agent: (**a**) Tensile Strength; (**b**) Flexural Strength; (**c**) Elasticity Modulus; and (**d**) Impact Strength.

Figure 7a–d shows the flexural strength, tensile strength, elasticity modulus, and impact strength of wood fiber/HDPE composites at different drying conditions. The results are given as averages and standard deviations (in parentheses) from the mean values. The mechanical properties of the composites with high-temperature hot air (HTHA)-treated wood fibers were improved as the temperature increased from 160 °C to 220 °C. The mechanical strength reached the highest value at a temperature of 220 °C. Compared with the 160 °C HTHA treatment, the mechanical strength of 220 °C HTHA treatment was increased by more than 20%. The relative crystallinity of cellulose and the relative amount of lignin increased during the treatment due to the partial pyrolysis of hemicellulose, and thus the elasticity modulus improved. Nevertheless, the flexural strength, elasticity modulus, and impact strength decreased dramatically at 240 °C. This indicated that a further increase of temperature resulted in more pyrolysis inside the cellulose. The increase of the cellulose chain ruptures led to decreased mechanical properties. With the increase of air velocity, flexural strength, tensile strength, and elasticity modulus of wood fiber/HDPE composites showed a trend of first increasing and then subsequent decreasing. This was because, at a lower air volume, the fiber had longer retention in high-temperature hot air, which caused more thermolysis in the fibers and the decomposition of hemicellulose and cellulose [33]. The damages of the wood fibers resulted in decreased mechanical properties of wood fiber/HDPE composites. However, the fiber had a short retention in the hot air when the treatment system operated at 13 m/s. Before a complete reaction, the hydroxide radicals in the fiber finished the heat treatment process, which caused a decrease of the mechanical properties. At different feed rates, the mechanical properties of the composites changed irregularly.

### 3.7. Dynamic Mechanical Analysis

**Figure 8:**
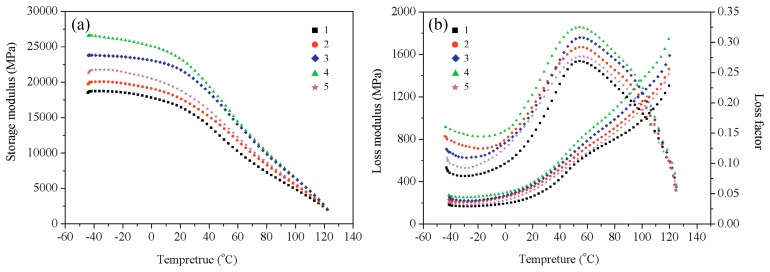
Variation of (**a**) the storage modulus and (**b**) the loss modulus and the loss factor with the steam temperature: (1) *T* = 160 °C; (2) *T* = 180 °C; (3) *T* = 200 °C; (4) *T* = 220 °C; and (5) *T* = 240 °C.

The storage moduli of wood fiber/HDPE composites at different high-temperature hot air (HTHA) treatment temperature are shown in Figure 8a. The storage modulus of the wood fiber/HDPE composites increased with increasing temperature. When the fibers were modified through exposure to high-temperature hot air at a temperature of 220 °C, the resulting wood fiber/HDPE composites showed the highest storage moduli.

Figure 8b reveals the variation of the loss modulus and the loss factor of the prepared wood fiber/HDPE composites as a function of temperature. The peaks in these two curves have been used to identify the glass-transition temperature (*T*_g_), and use the temperature corresponding to the maximum loss modulus as *T*_g_, which corresponds to the temperature at which the polymer chain begins to move or freeze [34]. In this experiment, the increase of *T*_g_ indicates that the length of the HDPE resin molecular chains is limited and that high-temperature modification can improve the compatibility between the wood fibers and the HDPE resin. Figure 8b demonstrates that the loss modulus curves obtained for the wood fiber/HDPE composites prepared from modified fibers are all single-peak curves, indicating that the modification leads to a good compatibility between the wood fibers and the HDPE. When the processing temperature was 220 °C, the loss modulus of the WPCs prepared from the modified fibers acquired the maximum value.

As mentioned above, the interfacial bonding between the wood fibers and the polymer matrix can also be evaluated using the adhesion factor (*A*). Strong interactions between the fibers and polymer matrix at the interface tend to reduce the macromolecular mobility in the vicinity of the filler surface compared to that in the bulk matrix [35]. The *A* values for the WPCs at 40 °C are calculated by using Equation (2). The wood fiber/HDPE composites modified by HTHA treatment at 160 °C exhibited the highest *A* values of 0.731 (the weakest interfacial interaction); however, the wood fiber/HDPE composites prepared from fibers modified by HTHA treatment (220 °C) exhibited the strongest interactions (lowest *A* value: 0.513). This result demonstrated that high-temperature hot air strongly affected the interfacial bonding between the wood fibers and the polymer matrix, and also the properties of the prepared composites.

### 3.8. Moisture Adsorption Characteristics

**Figure 9:**
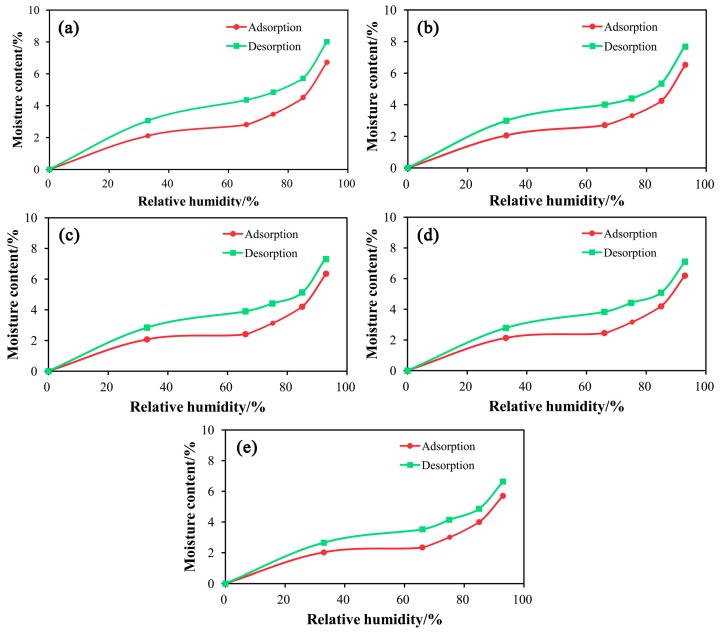
Comparative analysis of the adsorption and desorption behavior of the prepared composites containing different mass percentages of modified poplar wood fibers: (**a**) *T* = 160 °C; (**b**) *T* = 180 °C; (**c**) *T* = 200 °C; (**d**) *T* = 220 °C; (**e**) *T* = 240 °C.

Table 5 and Table 6 show the equilibrium moisture contents (EMCs) of the high-temperature hot air treated wood fiber /HDPE composites when they reached the adsorption and desorption equilibrium, respectively, under different relative humidity conditions. Figure 9 suggests that increasing the temperature of HTHA led to the decrease of moisture absorption of the HDPE/wood fiber composites. Under the same humidity conditions, the EMC reached through adsorption was lower than that reached through desorption, indicating the presence of an adsorption hysteresis phenomenon [36]. When the temperature of HTHA increased, the EMC of the composite decreased. The water adsorption isotherm obtained for the HTHA treated wood fiber/HDPE composites resembles a sigmoid shaped curve, as shown in Figure 9. Sigmoid shaped curve belongs to one type of six solid moisture absorption curves. In the range of 0% to 33% relative humidity, the absorption is in monomolecular type and it is easy to be performed. When the relative humidity is from 33% to 66%, hydrogen bonding occurs between the water molecule entering the wood fiber and water molecule adsorbed on the wood fiber, thus forming the state of “multi-molecular layer sorption”. When the relative humidity is in the range of 66% to 93%, the capillary condensed water appears, and with the increase of relative humidity, the proportion of capillary condensed water in the sorption increases. The increasing temperature of HTHA treatment of wood fiber effectively decreased the water desorption of the WPCs. This result contributed to the reduced shrinkage rate and the enhanced dimensional stability of the composite. Therefore, the number of surface cracks reduced and dimensional stability and the mechanical performance of the composite was reinforced.

### 3.9. Scanning Electron Microscopy (SEM)

**Figure 10:**
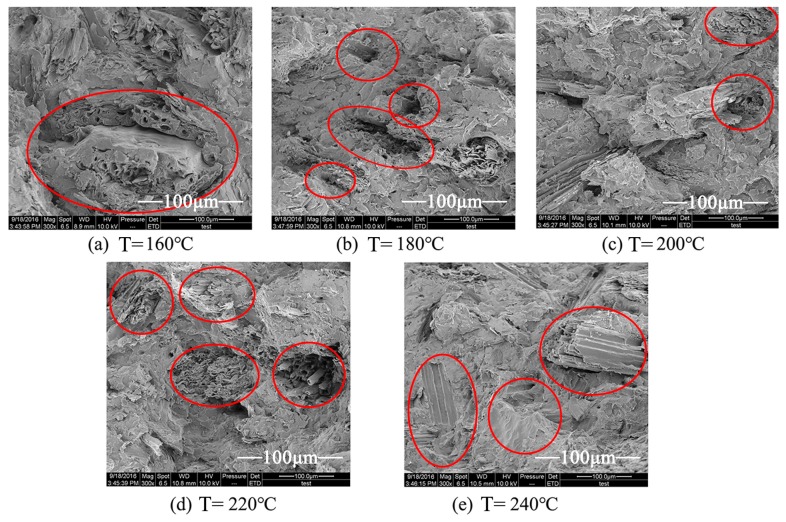
Surface morphology of the composite samples after high-temperature hot air treatment (magnification 300×) under (**a**) *T* = 160 °C; (**b**) *T* = 180 °C; (**c**) *T* = 200 °C; (**d**) *T* = 220 °C; and (**e**) *T* = 240 °C.

Figure 10 shows the SEM micrographs of wood fibers in WPCs after HTHA treatment and silane coupling modification. The results showed that the wood fibers were well-wrapped by the plastic after high-temperature hot air (HTHA) treatment, which indicated that HTHA treatment and silane coupling agent modification improved dispersion and compatibility between the fiber and plastic matrix, and further enhanced their interfacial binding in the composite material. Consequently, the material absorbed more energy under a continuous force with a clear enhancement of the mechanical properties, plasticity, and ductility of the composite material. Factors contributing to this improvement included the wood fiber in WPCs containing some polar groups, such as alcoholic hydroxyl and phenolic hydroxyl groups [37]. Furthermore, HTHA treatment and silane coupling agent modification decreased the number of alcoholic hydroxyl and phenolic hydroxyl groups, which improved the interfacial compatibility between the wood fiber and plastic, and consequently the mechanical properties of the composite material.

Figure 10a shows the fractured surface of the WPCs prepared under a temperature of 160 °C. It was observed that a large number of agglomerated fibers were found on the impact-fractured surfaces. This suggested that the hydroxyl groups on the bridging part of wood fibers under 160 °C were dehydrated to form a chemical bond, which made the wood fibers agglomerate, leading to the poor dispersion of wood fibers in the resin matrix and very low interaction between these two components. Although agglomeration of wood fibers was not clear on the fractured surface, the pull-out of fibers occurred more frequently, which resulted in a large number of voids on the fractured surface as illustrated in Figure 10b. Figure 10c,d showed that the wood fibers were well-wrapped, indicating that the interfacial bonding between the modified wood fibers and the plastic matrix significantly improved. When the fibers were treated at a temperature of 200 °C or 220 °C, the polarity of the fibers decreased, which contributed to the improved interfacial bonding between wood fibers and plastic matrix. These results indicated that the prepared WPCs showed better mechanical properties, as revealed in Figure 10d. Partial tearing was observed on the surface of the fibers, indicating that the brittleness of the wood fibers increased under 240 °C in Figure 10e. 

## 4. Conclusions

High-temperature hot air (HTHA) treatment and silane coupling agent were conducted for poplar fibers used in WPCs. The effects of HTHA treatment (i.e., temperature, inlet air velocity, feed rate) on the quality of modified poplar wood fibers and the properties of resultant composites were investigated. The results can be summarized as follows:

FTIR results indicated that the silane (A-187) and poplar wood fibers formed an effective chemical bond to enhance HDPE/wood fiber composites. The number of hydroxyl and carbonyl groups was reduced significantly with increasing temperature of HTHA, which decreased fiber polarity and resulted in a better compatibility between the fiber and plastic matrix. 

After modification of HTHA and the silane coupling agent, the number of hydroxyl groups on the surface of the fibers and surface polarity decreased, whereas the crystallinity and thermal stability significantly increased. When the temperature of HTHA was 240 °C, the crystallinity of cellulose increased to 68%, an increase of 28% compared to poplar wood fibers at a HTHA temperature of 160 °C. Moreover, with increasing temperature of HTHA, the storage modulus and mechanical properties significantly improved. At a HTHA temperature of 220 °C, the storage modulus and the loss modulus of the composite both reached an optimum.

The equilibrium moisture content of the composite along the adsorption and the desorption direction both increased, whereas the equilibrium moisture content along the adsorption direction was less than that along desorption direction, resulting in a moisture hysteresis.

HTHA and silane coupling agent-treated fibers had good dispersibility and stability during the mixing or compounding process. Selecting an appropriate HTHA and silane coupling agent modification method is of significance for the performance of wood fibers and WPCs reinforced with fiber materials. Compared with the lower temperature condition of HTHA, the quality of fibers treated by HTHA at higher temperature, the composite material showed superior performance. With the increasing temperature of HTHA, the thermal stability of the wood fiber significantly improved; however, the holocellulose content and the pH of wood fibers gradually decreased.

## Figures and Tables

**Table 1:** Characteristics of Poplar Wood Fibers.

Property Measured	Charateristics
Fiber morphology (mesh)	60 to 80
Fiber length (mm)	1.36 (0.20)
Fiber diameter (μm)	223 (31)
Length-diameter Ratio	6.1 (1.87)
MC at 23 °C (%)	12.4 (0.19)

Note: The results are given as averages and standard deviations (in parentheses) from the mean values; MC = Moisture Content.

**Table 2:** FTIR Spectra Attribution Results.

Wavenumber (cm^−1^)	Spectra Attribution
3350	Hydroxyl (–OH) stretching vibration
2890	Methyl (–CH_3_), methylene (=CH_2_) stretching vibration
1730	Carbonyl (C=O) stretching vibration in xylan (hemicellulose)
1600	Benzene ring carbon skeleton stretching (lignin)
1240	Benzene-oxygen bond stretching vibration (lignin); acyl-oxygen bond (CO–OR) stretching vibration (hemicellulose)
1030	Secondary alcohols and aromatic ether (C–O) stretching vibration (cellulose, hemicellulose, and lignin)
767	Si–O stretching vibrations

**Table 3:** Residence time of poplar wood fibers with different conditions of high-temperature hot air treatment.

Condition 1	Temperature (°C)	160	180	200	220	240
	Residence time (s)	19.3 ± 2.3	19.1 ± 1.6	19.0 ± 2.5	18.6 ± 2.1	18.1 ± 1.9
Condition 2	Inlet air velocity (m/s)	9	10	11	12	13
	Residence time (s)	31.2 ± 3.7	25.3 ± 2.9	19.0 ± 2.5	12.4 ± 3.2	7.9 ± 2.1
Condition 3	Feed rate (kg/h)	90	105	120	135	150
	Residence time (s)	12.1 ± 3.2	17.1 ± 3.1	19.0 ± 2.5	20.7 ± 2.6	21.1 ± 4.7

Note: (Condition 1) different temperatures with constant inlet air velocity 11 m/s and feed rate 120 kg/h. (Condition 2) different inlet air velocities with constant temperature 200 °C and feed rate 120 kg/h. (Condition 3) different feed rates with constant temperature 200 °C and inlet air velocity 11 m/s.

**Table 4:** Air flow humidity of air flow with different conditions of high-temperature hot air treatment.

Condition 1	Temperature (°C)	160	180	200	220	240
	Air flow humidity (g·kg^−1^)	38 ± 3.7	39 ± 4.3	42 ± 5.7	45 ± 5.8	49 ± 6.1
Condition 2	Inlet air velocity (m/s)	9	10	11	12	13
	Air flow humidity (g·kg^−1^)	75 ± 7.1	63 ± 6.4	42 ± 5.7	37	35
Condition 3	Feed rate (kg/h)	90	105	120	135	150
	Air flow humidity (g·kg^−1^)	31 ± 3.4	37 ± 4.3	42 ± 5.7	66 ± 6.7	73 ± 6.8

Note: (Condition 1) different temperatures with constant inlet air velocity 11 m/s and feed rate 120 kg/h. (Condition 2) different inlet air velocities with constant temperature 200 °C and feed rate 120 kg/h. (Condition 3) different feed rates with constant temperature 200 °C and inlet air velocity 11 m/s.

**Table 5:** Comparison of the EMCs of the composites along the moisture adsorption direction.

RH/%	Sample 1 (160 °C mPWF)	Sample 2 (180 °C mPWF)	Sample 3 (200 °C mPWF)	Sample 4 (220 °C mPWF)	Sample 5 (240 °C mPWF)
33	2.05 ± 0.18	2.01 ± 0.19	1.99 ± 0.16	1.98 ± 0.13	1.93 ± 0.14
66	2.75 ± 0.19	2.65 ± 0.18	2.32 ± 0.17	2.28 ± 0.15	2.21 ± 0.15
75	3.51 ± 0.24	3.49 ± 0.23	3.35 ± 0.22	3.23 ± 0.23	3.08 ± 0.20
85	4.45 ± 0.29	4.19 ± 0.25	4.11 ± 0.24	4.02 ± 0.25	3.86 ± 0.24
93	6.66 ± 0.38	6.47 ± 0.34	6.26 ± 0.37	6.03 ± 0.38	5.57 ± 0.33

**Table 6:** Comparison of the EMCs of the composites along the desorption direction.

RH/%	Sample 1 (160 °C mPWF)	Sample 2 (180 °C mPWF)	Sample 3 (200 °C mPWF)	Sample 4 (220 °C mPWF)	Sample 5 (240 °C mPWF)
33	3.01 ± 0.20	2.93 ± 0.21	2.75 ± 0.21	2.61 ± 0.18	2.56 ± 0.18
66	4.30 ± 0.28	3.95 ± 0.23	3.81 ± 0.23	3.66 ± 0.21	3.43 ± 0.22
75	4.92 ± 0.34	4.34 ± 0.22	4.32 ± 0.23	4.25 ± 0.21	4.06 ± 0.25
85	5.65 ± 0.33	5.29 ± 0.34	5.04 ± 0.34	5.01 ± 0.30	4.78 ± 0.30
93	7.98 ± 0.46	7.41 ± 0.40	7.23 ± 0.42	6.93 ± 0.39	6.54 ± 0.38
